# Supplementary figures and images for: Towards a distributed connectionist account of cognates and interlingual homographs: evidence from semantic relatedness tasks
Source: PeerJ. 2019 May 16;7:e6725. doi: 10.7717/peerj.6725 (PMC6526012; doi:10.7717/peerj.6725)

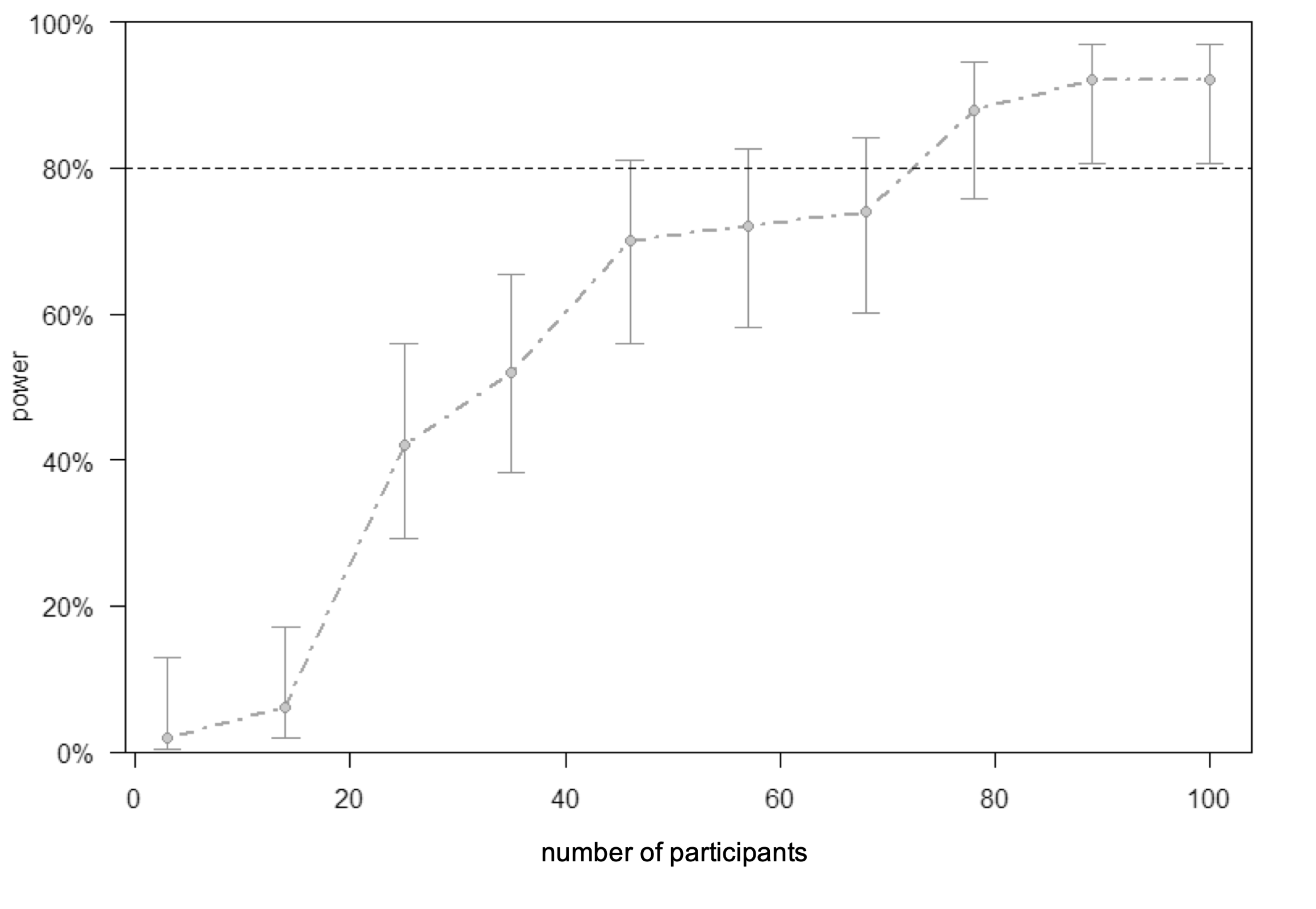

Supplement: Figure S1 — The level of power is displayed on the y-axis. The number of participants required to achieve a certain level of power is displayed on the x-axis. The dashed black line indicates 80% power. The simulation calculated the average estimated level of power (and 95% confidence interval) for a smallest-effect-size-of-interest of approximately 20 ms for 10 sample sizes between 0 and 100, based on 50 simulations each. [file peerj-07-6725-s001.png]

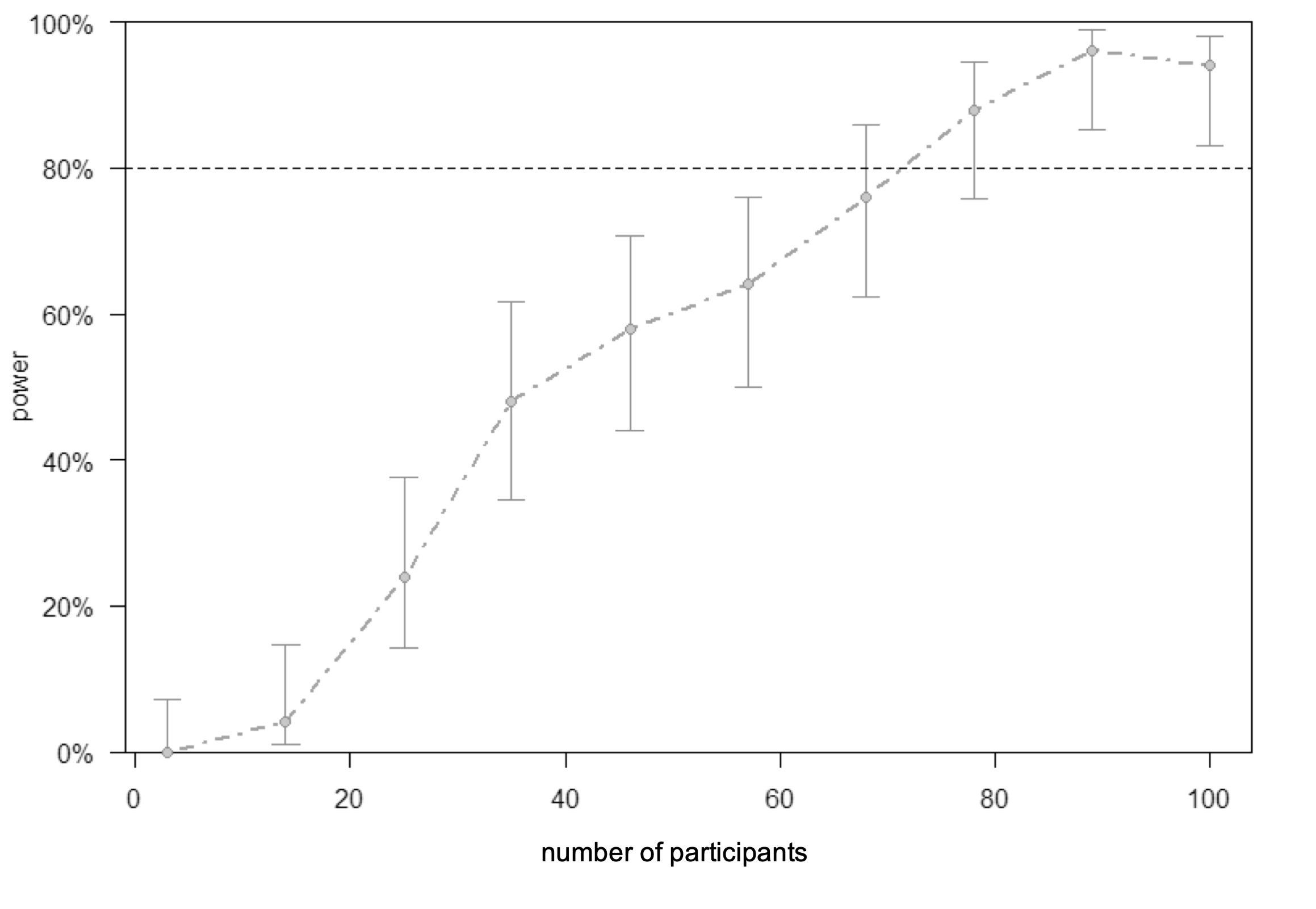

Supplement: Figure S2 — The level of power is displayed on the y-axis. The number of participants required to achieve a certain level of power is displayed on the x-axis. The dashed black line indicates 80% power. The simulation calculated the average estimated level of power (and 95% confidence interval) for a smallest-effect-size-of-interest of approximately 20 ms for 10 sample sizes between 0 and 100, based on 50 simulations each. [file peerj-07-6725-s002.png]

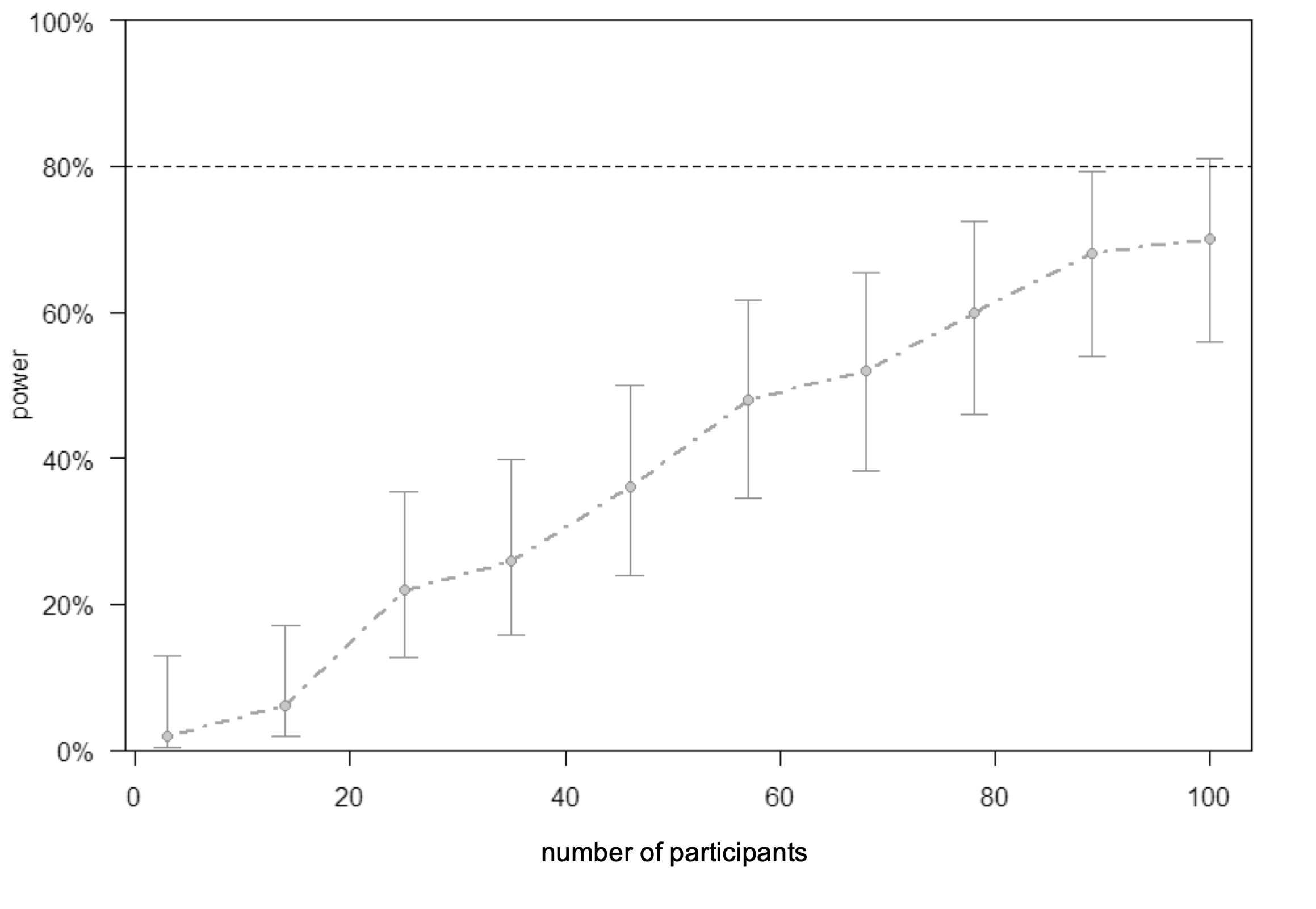

Supplement: Figure S3 — The level of power is displayed on the y-axis. The number of participants required to achieve a certain level of power is displayed on the x-axis. The dashed black line indicates 80% power. The simulation calculated the average estimated level of power (and 95% confidence interval) for a smallest-effect-size-of-interest of approximately 20 ms for the cognates and approximately 0 ms for the translation equivalents for 10 sample sizes between 0 and 100, based on 50 simulations each. [file peerj-07-6725-s003.png]

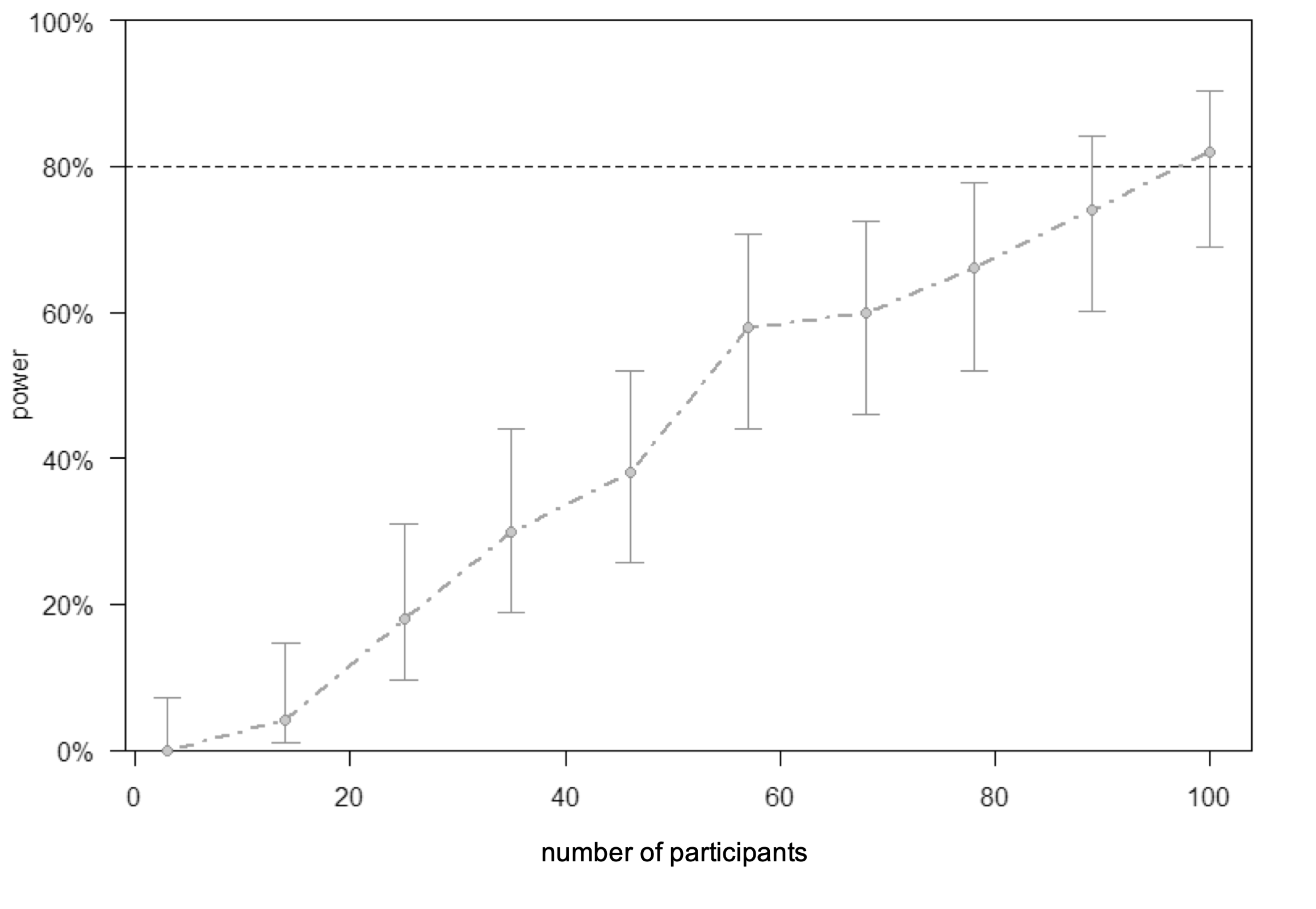

Supplement: Figure S4 — The level of power is displayed on the y-axis. The number of participants required to achieve a certain level of power is displayed on the x-axis. The dashed black line indicates 80% power. The simulation calculated the average estimated level of power (and 95% confidence interval) for a smallest-effect-size-of-interest of approximately 20 ms for the interlingual homographs and approximately 0 ms for the translation equivalents for 10 sample sizes between 0 and 100, based on 50 simulations each. [file peerj-07-6725-s004.png]
